# C/EBPα induces *Ebf1* gene expression in common lymphoid progenitors

**DOI:** 10.1371/journal.pone.0244161

**Published:** 2020-12-17

**Authors:** Theresa Barberi, Cheng Cui, Alan D. Friedman

**Affiliations:** 1 Division of Pediatric Oncology, Johns Hopkins University School of Medicine, Baltimore, Maryland, United States of America; 2 Department of Physiology, China Medical University, Shenyang, China; St. Vincent's Institute, AUSTRALIA

## Abstract

C/EBPα is required for formation of granulocyte-monocyte progenitors (GMP) and also participates in B lymphopoiesis. The common lymphoid progenitor (CLP) and preproB populations but not proB cells express *Cebpa*, and pan-hematopoietic deletion of the +37 kb *Cebpa* enhancer using Mx1-Cre leads not only to reduced GMP but also to 2-fold reduced marrow preproB and >15-fold reduced proB and preB cells. We now show that IL7Rα-Cre-mediated deletion of the +37 kb *Cebpa* enhancer, which occurs in 89% of Ly6D^+^ and 65% of upstream Ly6D^-^ CLP, leads to a 2-fold reduction of both preproB and proB cells, and a 3-fold reduction in preB cells, with no impact on GMP numbers. These data support a direct role for C/EBPα during B lineage development, with reduced enhancer deletion in Ly6D^-^ CLP mediated by IL7Rα-Cre diminishing the effect on B lymphopoiesis compared to that seen with Mx1-Cre. Amongst mRNAs encoding key transcriptional regulators that initiate B lymphoid specification (PU.1, E2A, IKAROS, EBF1, FOXO1, and BACH2), only *Ebf1* levels are altered in CLP upon Mx1-Cre-mediated *Cebpa* enhancer deletion, with *Ebf1* reduced ~40-fold in Flt3^+^Sca-1^int^c-kit^int^IL7Rα^+^ CLP. In addition, *Cebpa* and *Ebf1* RNAs were 4- and 14-fold higher in hCD4^+^ versus hCD4^-^ CLP from *Cebpa*-hCD4 transgenic mice. Histone modification ChIP-Seq data for CLP indicate the presence of active, intronic *Ebf1* enhancers located 270 and 280 kb upstream of the transcription start sites. We identified a *cis* element in this region that strongly binds C/EBPα using the electrophoretic mobility shift assay. Mutation of this C/EBPα-binding site in an *Ebf1* enhancer-TK-luciferase reporter leads to a 4-fold reduction in C/EBPα-mediated trans-activation. These findings support a model of B lymphopoiesis in which induction of *Ebf1* by C/EBPα in a subset of CLP contributes to initiation of B lymphopoiesis.

## Introduction

CCAAT/enhancer binding protein α (C/EBPα) is a basic region-leucine zipper transcription factor expressed prominently in hepatocytes, adipocytes, pulmonary type II pneumocytes, and hematopoietic myeloid cells [1–3]. C/EBPα is required for the formation of the granulocyte-monocyte progenitor (GMP) in adult bone marrow and thus also for the generation of mature neutrophils, monocytes, and eosinophils [4, 5].

In addition to its central role in myelopoiesis, C/EBPα contributes to early B lymphoid development. The murine *Cebpa* gene contains a 450 bp enhancer, located at +37 kb, that is active specifically in hematopoietic cells [6–8]. In particular, cytomegalovirus (CMV)-Cre-mediated deletion of the *Cebpa* +37 kb enhancer leads to 28-fold reduced marrow *Cebpa* mRNA without affecting expression in liver, adipose, or lung, and the corresponding, highly conserved, human +42 kb enhancer contains activating histone marks in hematopoietic myeloid cells but not in other tissues [9, 10]. Unlike deletion of the *Cebpa* open-reading frame, which generally leads to death prior to one month of age due bacterial infection consequent to severe neutropenia, the inducible Mx1-Cre-mediated deletion of the hematopoietic-specific +37 kb enhancer allows maintenance of peripheral neutrophil counts at a level compatible with survival for at least 18 months [5]. Upon Mx1-Cre-mediated deletion of the +37 kb *Cebpa* enhancer, *Cebpa* mRNA is 14-fold reduced in GMP and 8-fold decreased in the common lymphoid progenitor (CLP) population, with 4-fold fewer B-lymphoid colonies forming in cultures with IL-7, 2-fold reduced marrow preproB cells, and >15-fold reduction of marrow proB, early preB, and preB cells [9, 11]. In contrast, splenic and thymic T cell numbers are unaffected by *Cebpa* enhancer deletion [11].

The mechanism whereby C/EBPα contributes to B lymphopoiesis remains uncertain. In one model of hematopoiesis, Flt3^-^CD34^-^ long-term and Flt3^-^CD34^+^ short-term hematopoietic stem cells give rise to Flt3^+^CD34^+^ lymphoid-primed multipotent progenitors (LMPP), which in turn generate GMP and CLP, the latter having B and T lymphoid, natural killer (NK), and dendritic cell (DC) potential. Onset of interleukin-7 receptor α (IL7Rα) expression with diminution of Sca-1 and c-kit broadly defines the Lineage-negative (Lin^-^)Sca-1^int^c-kit^int^IL7Rα^+^Flt3^+^ CLP population, from which preproB and then proB cells develop [12]. CLP can be further divided into an Ly6D^-^ multipotent subset and an Ly6D^+^ unipotent subset, the latter mainly generating B cells *in vivo* [13]. During normal B cell development, IKAROS and PU.1 induce E2A/TCF3 expression, which in turn activates the genes encoding EBF1 and FOXO1; EBF1 and FOXO1 then generate a positive-feedback loop and stimulate PAX5 expression to initiate proB cell formation followed by downstream B lineage development [14–19]. E2A also induces BACH2, which stimulates FOXO1 expression to favor B lymphopoiesis while inhibiting myelopoiesis [20]. IKAROS (encoded by *Izkf1*) and PU.1 are required in LMPP to initiate lymphoid development, E2A is required for progression beyond the Ly6D^-^ CLP stage, and EBF1 and FOXO1 are first expressed above minimal levels and specify B lymphopoiesis in Ly6D^+^ CLP [13–17].

We previously found that *Cebpa* +37 kb enhancer deletion using Mb1-Cre, which is initially expressed at the proB stage, does not affect B lineage development [11]. We now find that deletion of the *Cebpa* enhancer with IL7Rα-Cre, but not Rag1-Cre, impairs B lymphopoiesis without inducing the reduction of GMP or expansion of LMPP seen with pan-hematopoietic, Mx1-Cre-mediated enhancer deletion [9], providing support for a direct role for C/EBPα at an early stage of B cell development. IL7Rα-Cre deleted the STOP codon in a ROSA26-*lox*P-STOP-*lox*P-YFP (R26-LSL-YFP) allele in 89% of Ly6D^+^ CLP, but only in 65% of Ly6D^-^ CLP, and Rag1-Cre is less active in these populations. The reduced activity of IL7Rα-Cre in Ly6D^-^ CLP, together with a lag in enhancer excision, may account for the more modest effect on B lymphopoiesis of IL7Rα-Cre- compared with Mx1-Cre-mediated *Cebpa* enhancer deletion.

Examination of our prior microarray data reveals that *Cebpa* and *Ebf1* mRNA levels are each reduced 4-fold in preproB cells upon Mx1-Cre-mediated *Cebpa* +37 kb enhancer deletion, whereas *Izkf1*, *E2A*, *Foxo1*, and *Bach2* levels are unchanged. We now further find that *Cebpa* enhancer deletion essentially eliminates *Ebf1* expression in CLP, whereas *Pu*.*1*, *Izkf1*, *E2A*, *Foxo1*, and *Bach2* levels are unaffected. In addition, RNA analysis of CLP sorted from *Cebpa* +37 kb enhancer/promoter-hCD4 transgenic reporter mice reveals 4-fold increased *Cebpa* and 14-fold increased *Ebf1* expression in the hCD4^+^ compared with the hCD4^-^ population. Through analysis of available ChIP-Seq data, we have identified an evolutionarily conserved *Ebf1* +270/280 kb genomic region that contains activating H3K4me1 and H3K27Ac histone marks, indicative of potential *Ebf1* enhancers, as well as several sites that match or nearly match the C/EBPα DNA-binding consensus within evolutionarily conserved sub-domains of this region. An oligonucleotide containing one of these consensus sites binds C/EBPα strongly in the gel shift assay, and mutation of this site leads to 4-fold reduced C/EBPα-mediated trans-activation of an *Ebf1* enhancer-luciferase reporter. Together, our findings support a model wherein C/EBPα induces *Ebf1* expression in CLP to facilitate B lymphopoiesis.

## Materials and methods

### Ethics statement

This study was carried out in strict accordance with the recommendations in the Guide for the Care and Use of Laboratory Animals of the National Institutes of Health. The protocol (M019M10) was approved by the Johns Hopkins University Animal Care and Use Committee. All efforts were made to minimize suffering. As procedures caused only momentary, slight pain analgesics and anesthesia were not required. Euthanasia prior to marrow removal was by carbon dioxide asphyxiation, followed by cervical dislocation.

### Murine models

*Cebpa* Enh(f/f) and Enh(f/f);Mx1-Cre mice, having *lox*P sites surrounding the +37 kb *Cebpa* enhancer, were described previously [9]. Enhancer deletion was induced in Enh(f/f);Mx1-Cre mice by intra-peritoneal injection of 300 μg of pIpC (Invivogen) every other day for three doses. Rag1-Cre, IL7Rα-Cre, and R26-LSL-YFP mice (Jackson Laboratory, #006148) were previously described [21–23]. *Cebpa* Enh/Prom-hCD4 mice possess a transgene linking the +37 kb *Cebpa* enhancer to the *Cebpa* promoter and a human CD4 (hCD4) reporter [7]. All alleles were on the C57BL/6 background. Mice were housed in ventilated cages with a 12 hr light/dark cycle, maximum five mice per cage, and were provided acidified water and autoclaved Envigo 2018SX feed. Male and female 12- to 24-week old mice were utilized.

### Flow cytometry

Marrow was obtained by crushing femurs, tibia, and hips in phosphate-buffered saline (PBS) with 3% heat-inactivated fetal bovine serum (HI-FBS) using a mortar and pestle, then passage through a 40 μm cell strainer, followed by red blood cell lysis with ammonium chloride and enumeration of total mononuclear cells using a hemocytometer. After antibody staining, cell analysis or cell sorting were accomplished using an LSRFortessa analyzer or the FACSAria II sorter (BD Biosciences). Antibodies were from BD Biosciences unless otherwise indicated. For enumeration of B cell precursors, marrow was subjected to lineage-depletion by staining with biotin-anti-Lineage Cocktail (with anti-Gr1, -CD11b, -Ter119, and -CD3 but without anti-B220), biotin-anti-IgM (II/41, Invitrogen), and biotin-anti-NK1.1 (PK136, Invitrogen), followed by incubation with anti-biotin microbeads and passage through LD columns (Miltenyi). Resulting Lin^-^ cells were stained with anti-B220-APC (RA3-6B2, Biolegend), anti-CD43-BV786 or -PE (S7), anti-CD93-BV650, anti-BP-1-PE (Biolegend) or -FITC (6C3, Invitrogen), anti-CD24-BUV496 or -PE-Cy7, and anti-CD19-BV421. LMPP and CLP were identified using Lineage Cocktail (with anti-B220)-FITC (Biolegend), -BV421 (Biolegend), or -PerCP-Cy5.5, anti-Sca-1-PE-Cy7 (D7, Invitrogen), anti-c-kit-APC (2B8), anti-CD127-BV650 (A7R34, Biolegend), anti-Flt3-PE (A2F10.1), and anti-Ly6D-eF450 (49-4H, Invitrogen). YFP was detected in the FITC channel. Human CD4 was detected using anti-hCD4-BV785 (RPA-T4, Biolegend). Marrow was lineage-depleted prior to further staining and sorting for LMPP and CLP.

### RNA analysis

RNA from hematopoietic cells was prepared using NucleoSpin RNA II, with use of RNase-free DNase (Machery-Nagel). First strand cDNA was prepared using ImProm-II reverse transcriptase (Promega) and oligodT primer at 42°C for 1 hr. Quantitative PCR was carried out in triplicate using 5–25 ng of each cDNA using Radiant LoRox SYBR Green supermix (Alkali Scientific). *Cebpa*, *Ebf1*, *Pu*.*1*, *Pax5*, *Izkf1*, *E2A*, *Foxo1*, *Bach2*, and ribosomal subunit *mS16* internal control primers were:

Cebpa-F: 5’-TGGACAAGAACAGCAACGAG,

Cebpa-R: 5’-TCACTGGTCAACTCCAGCAC,

Ebf1-F: 5’-GGATACGGACAGAACAGGATTTC,

Ebf1-R: 5’-GGCACATTTCAGGGTTCTTGTC,

Pu.1-F: 5’- CCTTCGTGGGCAGCGATGGA,

Pu.1-R: 5’- TGTAGCTGCGGGGGCTGCAC,

Pax5-F: 5’-CTCTGACATCTTCACCACCAC,

Pax5-R: 5’-GTTGGCTTTCATGTCATCCAGG,

Izkf1-F: 5’-ATACAGAGAGCAACGCGGAG,

Izkf1-R: 5’-CGCTGCTCCTCCTTGAGAG,

E2A-F: 5’-TGATGTTCCCGCTACCTGTG,

E2A-R: 5’-CTTCGCTGTATGTCCGGCTA,

Foxo1-F: 5’-TCAAGGATAAGGGCGACAGC,

Foxo1-R: 5’-GCTCTTCTCCGGGGTGATTT,

Bach2-F: 5’-CCAAGTCCGACCCCAGATTA,

Bach2-R: 5’-GAAGTTTAACCTCCTGGCCC,

mS16-F: 5’-CTTGGAGGCTTCATCCACAT, and

mS16-R: 5’-ATATTCGGGTCCGTGTGAAG.

### Gel shift and chromatin immunoprecipitation assays

293T cells (ATCC, CRL-3216) were transiently transfected with 4μg CMV or 4 μg CMV-C/EBPα in 100 mm dishes using 15 μL Lipofectamine 2000 (Invitrogen). Nuclear extracts were prepared two days later and gel shift assay performed using 1 ng of radio-labelled probe and 6 μg of nuclear extract, as described [24]. Oligonucleotide probes containing 5′-GATC or 5’-TCGA overhangs were radio-labeled to similar specific activity using Klenow enzyme and α-P^32^-dCTP. Sense strands of the probes used, with binding sites underlined, were as follows:

Ebf1-α1: 5’-GATCCTGATAATGGAGGAAGAAATAAGCTAGCGG,

Ebf1-α2: 5’-GATCCAGTTTGCCTTTGAGTAATGTCGTCAATTTG,

Ebf1-α3: 5’-GATCGTTATTGTTAAAAGTTGGGCAAGGTTGAAATGC, and

NE-C/EBP: 5’-TCGAGGCCAGGATGGGGCAATACAACCCG.

Chromatin immunoprecipitation (ChIP) was conducted using 10 μL (1:50) rabbit monoclonal anti-C/EBPα antibody (Cell Signaling, #8178S) or normal rabbit immune globulin G (IgG, Cell Signaling #3900S) as described [25], with the use of the following genomic DNA PCR primer pairs:

Ebf1- α1F: 5’-CCCAGAAGTAAGGTGTACCAAGT,

Ebf1- α1R: 5’-CCAGCCTCCAGAGCAAAATC,

Ebf1- α2F: 5’-AGAGGCTCTTGCTATTTGAGCC,

Ebf1- α2R: 5’-ACTCAAGCCAAGTAACTCACCC,

Ebf1- α3F: 5’-GCGAGTTATTTGCAAAAGCGAA,

Ebf1- α3R: 5’-AGCTTTTGTACAAGCAGTTGGG,

PU.1enhF: 5’-CTGGTGGCAAGAGCGTTTC, and

PU.1enhR: 5’-CCACATCGGCAGCAGCAAG.

### Cell culture and transient transfection

The TK-Luc reporter, containing a minimal herpes simplex virus thymidine kinase (TK) promoter, was generated by ligating a double-stranded oligonucleotide between the *Not*I and *Hind*III sites of pREP4. The sequence of the sense strand, with TATAA box and RNA start sites underlined, is:

5’-TTCGCATATTAAGGTGACGCGTGTGGCCTCGAACACCGAGCGACCCTGCAG. 350 bp murine *Ebf1* genomic segments containing wild-type or mutant C/EBPα consensus site α3 were synthesized (Blue Heron) and ligated upstream between the *Kpn*I and *Not*I sites in plasmid TK-Luc to generate Ebf1-TK-Luc and mutant mEbf1-TK-Luc. 293T cells were cultured in Dulbecco’s modified Eagle medium with 10% FBS. 10^5^ cells/well were plated in 24 well dishes followed the next day by transient transfection using Lipofectamine 2000 with 75 ng of luciferase reporter plasmid, 10 ng CMV or CMV-C/EBPα, and 0.4 ng CMV-βGal. Total cell extracts were subjected to luciferase and β-galactosidase assays two days later, as described [8]. 32Dcl3 murine myeloid cells [26] were cultured in Iscove’s modified Dulbecco medium with 10% HI-FBS and 1 ng/mL murine interleukin-3 (Peprotech).

### Data analysis

Means and standard deviations (SD) are shown. The Student t test was used for statistical comparisons. Publicly available ChIP-Seq data were analyzed in the vicinity of the *Ebf1* gene for histone marks and C/EBPα binding (GSE60103 and GSE58362); publicly available CLP RNA-Seq data was analyzed for *Ebf1* and *Cebpa* expression (GSE92540); and our previous flow cytometry data (https://pubmed.ncbi.nlm.nih.gov/30061199/) was further analyzed for CLP sub-population numbers, as cited in the corresponding figure legends. The original image underlying gel shift results is provided (S1 Fig).

## Results

### Deletion of the *Cebpa* +37 kb enhancer using IL7Rα-Cre but not Rag1-Cre impairs B lineage development

**Fig 1:**
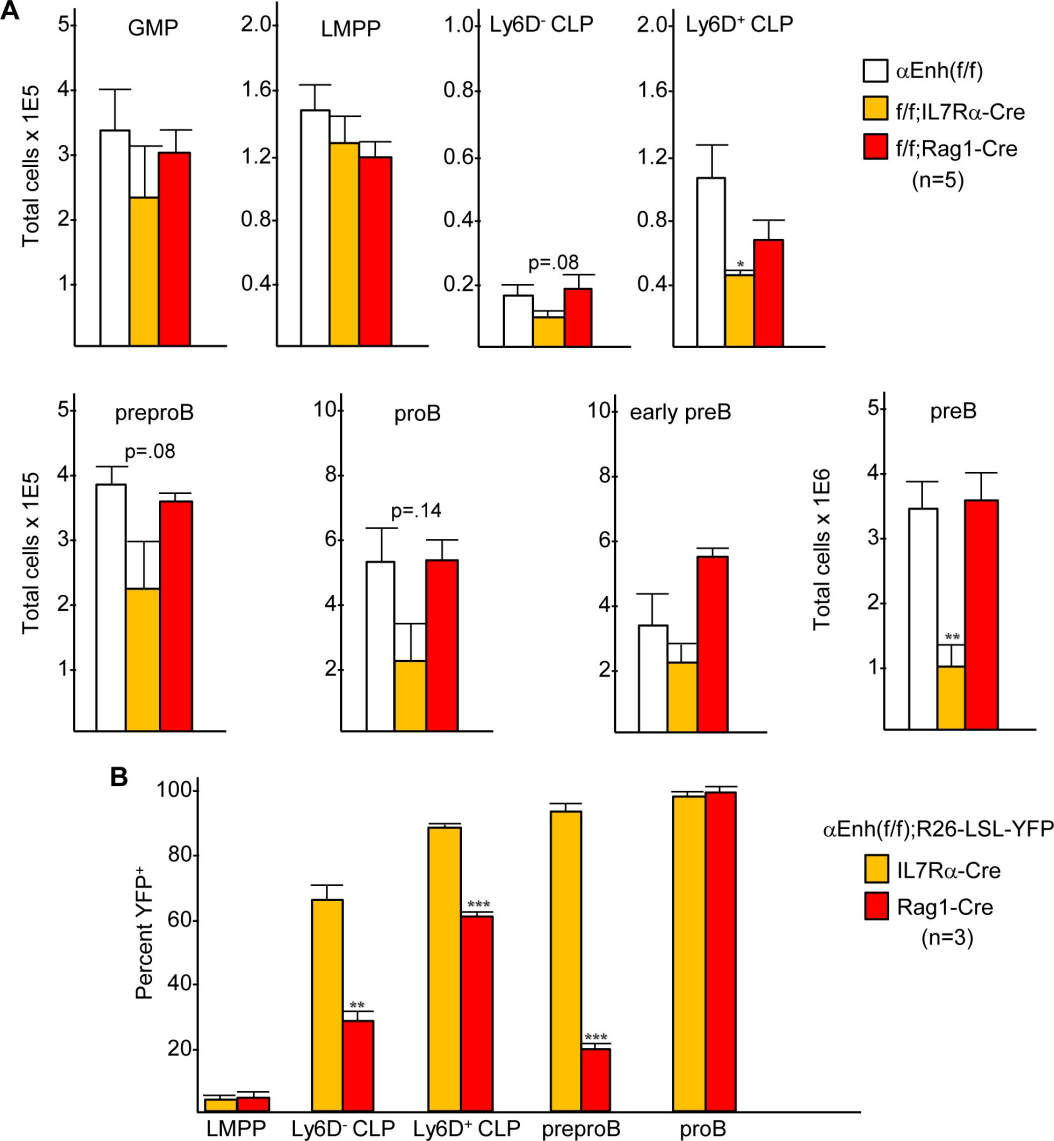
*Cebpa* +37 kb enhancer deletion using IL7Rα-Cre but not Rag1-Cre impairs B lymphopoiesis. **A**) Flow cytometry was used to enumerate GMP, LMPP, Ly6D^-^ and Ly6D^+^ CLP, preproB, proB, early preB, and preB cells in bone marrow isolated from bilateral leg and hip bones from *Cebpa* +37 kb Enh(f/f), Enh(f/f);IL7Rα-Cre, and Enh(f/f);Rag1-Cre mice (mean and SD from five determinations). **B**) Flow cytometry was used to determine the proportion of LMPP, Ly6D^-^ and Ly6D^+^ CLP, preproB, and proB cells from Enh(f/f);IL7Rα-Cre;R26-LSL-YFP or Enh(f/f);Rag1-Cre;R26-LSL-YFP mice that express YFP (mean and SD from three determinations). * p<0.05, ** p<0.01, *** p<0.001.

*Cebpa* Enh(f/f);Mx1-Cre mice exposed to pIpC have defects in both the myeloid and B lymphoid lineages, with expansion of upstream LMPPs. To further support a direct role for C/EBPα during B lymphopoiesis, we sought to specifically delete the +37 *Cebpa* enhancer in early B lineage progenitors. Rag1-Cre deletes the floxed stop codon in a R26-LSL-RFP allele in 3% of LMPP and 60% of CLP [21]. IL7Rα-Cre deletes the stop codon in R26-LSL-YFP in 10% of LMPP and 88% of CLP [22]. We bred these transgenes with *Cebpa* Enh(f/f) mice to generate Enh(f/f);IL7Rα-Cre and Enh(f/f);Rag1-Cre mice and evaluated their bone marrow GMP, LMPP, Ly6D^-^ and Ly6D^+^ CLP, preproB, proB, early preB and preB populations using flow cytometry (Fig 1A). Enh(f/f);Mx1-Cre mice exhibit several alterations: 2-fold reduced GMP and 6-fold increased LMPP [9]; >15-fold reduced proB, early preB, and preB cells, and 2-fold reduced preproB cells [11]; and 5-fold increased Lin^-^Sca-1^int^c-kit^int^IL7Rα^+^Flt3^-^ cells without alteration in Ly6D^-^ or Ly6D^+^ CLP (S2 Fig). As expected, Enh(f/f);IL7Rα-Cre mice had no significant change in marrow GMP or LMPP numbers; however, they did possess 2.5-fold reduced Ly6D^+^ CLP and a trend towards 2-fold reduced Ly6D^-^ CLP. Additionally, there was a 3.4-fold reduction in preB cells with a trend towards reduced preproB (1.7-fold) and proB cells (2.3-fold). In contrast, enhancer deletion using Rag1-Cre had minimal effects on these marrow populations.

To gain insight into the different effects on B lineage development resulting from *Cebpa* enhancer deletion in Enh(f/f);IL7Rα-Cre versus Enh(f/f);Rag1-Cre mice, we generated Enh(f/f);IL7Rα-Cre;R26-LSL-YFP and Enh(f/f);Rag1-Cre;R26-LSL-YFP mice and evaluated yellow fluorescence protein (YFP) expression as an indicator of Cre activity. Marrow was stained and analyzed for LMPP, Ly6D^-^ CLP, Ly6D^+^ CLP, preproB, and proB cells (Fig 1B), with representative flow cytometry plots shown (S3 Fig). Of note, preproB cells in our prior [11] and current analyses were depleted of NK cells but were not depleted of plasmacytoid dendritic cells, which express little *Cebpa* [27]. Consistent with prior findings, YFP was evident in less than 5% of LMPP in both models, and was expressed in a higher proportion of CLP from IL7Rα-Cre compared with Rag1-Cre mice. In both Enh(f/f);IL7Rα-Cre;R26-LSL-YFP and Enh(f/f);Rag1-Cre;R26-LSL-YFP mice, Ly6D was expressed, on average, in 74% of CLP. Rag1-Cre led to YFP expression in 27% of Ly6D^-^ and 61% of Ly6D^+^ CLP, whereas IL7Rα-Cre induced YFP expression in 65% of Ly6D^-^ and 89% of Ly6D^+^ CLP, on average. Both induced YFP in ~100% of proB cells, but IL7Rα-Cre led to YFP expression in 94% of the preproB population, compared with only 24% using Rag1-Cre. These data extend prior findings to show that IL7Rα-Cre is more active than Rag1-Cre in both Ly6D^+^ and Ly6D^-^ CLP; furthermore, we also find that IL7Rα-Cre only induces YFP in two-thirds of the Ly6D^-^ CLP subset, potentially reflecting both the point of onset of IL7Rα expression and delayed kinetics of floxed allele deletion. These findings may account, at least in part, for the quantitatively different B lineage alterations seen in Enh(f/f);Mx1-Cre, Enh(f/f);IL7Rα-Cre, and Enh(f/f);Rag1-Cre mice.

### Deletion of the *Cebpa* +37 kb enhancer using Mx1-Cre leads to markedly reduced *Ebf1* expression in CLP

Our prior global gene expression analysis of preproB cells from *Cebpa* Enh(f/f) versus *Cebpa* Enh(f/f);Mx1-Cre mice, which were administered pIpC four weeks prior to RNA isolation, reveals 4-fold reduction in both *Cebpa* and *Ebf1* mRNA expression, on average, with no change in *Flt3*, *IL7Rα*, *E2A*, *Izkf1*, *Foxo1*, *Bach2*, *Irf4*, or *Irf8* [11]. *Pu*.*1* was not detected on the array due to its low level at this stage of B cell development. The mean fluorescence intensity of surface Flt3 and IL7Rα, as evaluated by flow cytometry, was not reduced in CLP as a result of Mx1-Cre mediated enhancer deletion [9].

**Fig 2:**
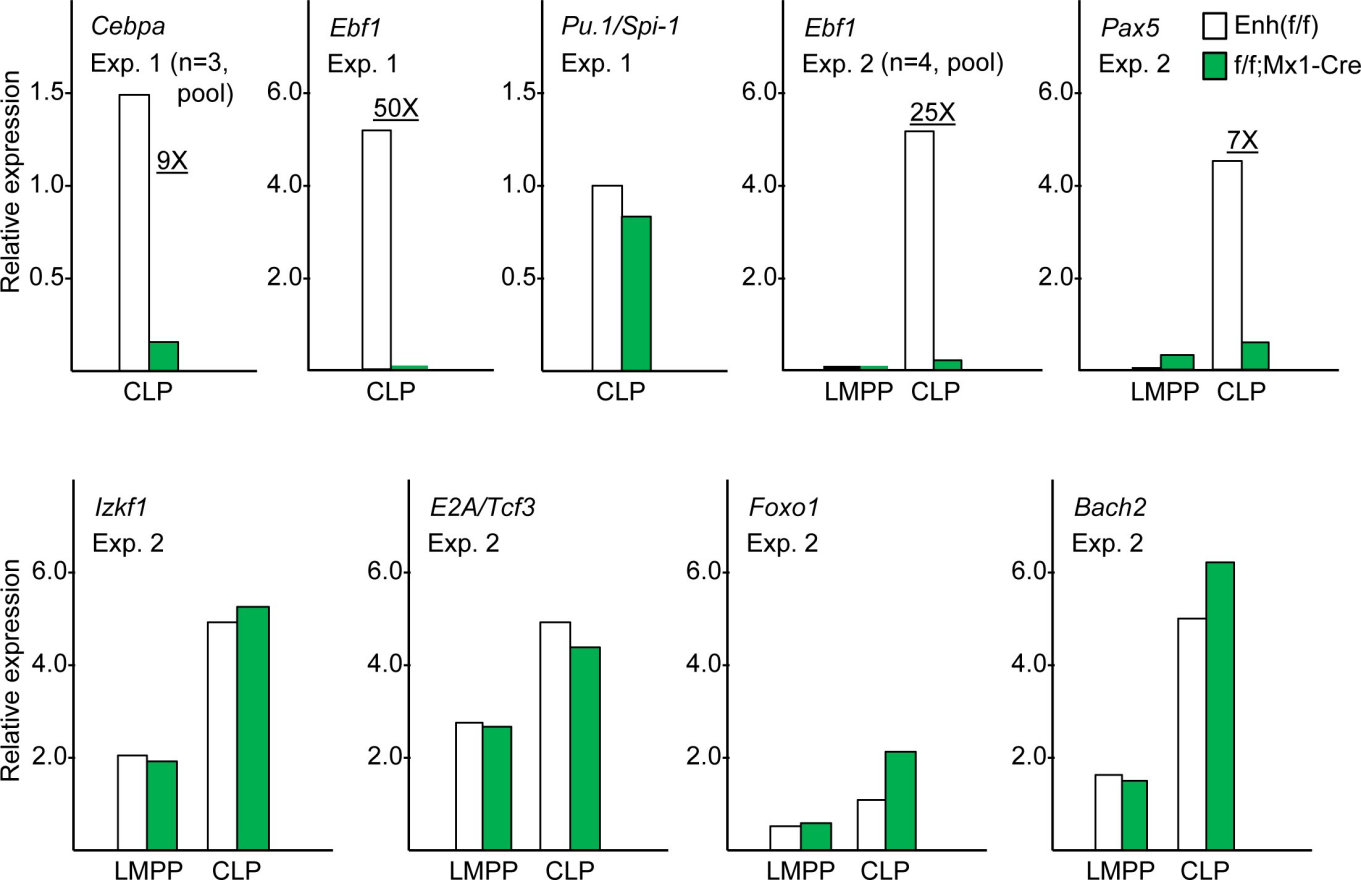
Deletion of the *Cebpa* +37 kb enhancer using Mx1-Cre leads to marked reduction of *Ebf1* mRNA in CLP. In Experiment 1, CLP sorted from three Enh(f/f) or Enh(f/f);Mx1-Cre mice were pooled and then analyzed by qRT-PCR for expression of *Cebpa*, *Ebf1*, or *Pu*.*1/Spi-1*. In Experiment 2, LMPP and CLP sorted from four Enh(f/f) or Enh(f/f);Mx1-Cre mice were pooled and then analyzed by qRT-PCR for expression of *Ebf1*, *Pax5*, *Izkf1*, *E2A/Tcf3*, *Foxo1*, and *Bach2*.

We evaluated RNA expression of *Ebf1* and additional B-lineage transcription factors in sorted CLP (experiments 1 and 2) and LMPP (experiment 2) from *Cebpa* Enh(f/f) and Enh(f/f);Mx1-Cre mice exposed 4 weeks earlier to pIpC. Enh(f/f);Mx1-Cre rather than Enh(f/f);IL7Rα-Cre mice were chosen for these analyses due to our finding that IL7Rα-Cre does not delete the enhancer in the entire CLP population. Consistent with prior findings, *Cebpa* was reduced 9-fold in CLP. In both experiments, each pooling CLP subsets or LMPP from several mice, deletion of the *Cebpa* +37 kb enhancer by Mx1-Cre led to a striking loss of *Ebf1* expression. In particular, *Ebf1* was reduced 25- or 50-fold in CLP. In contrast, levels of *Pu*.*1*, *Izkf1*, *E2A*, *Foxo1*, and *Bach2* were unchanged in CLP (Fig 2). *Pax5* mRNA was reduced 7-fold in CLP, reflecting regulation of its cognate gene by EBF1. Of note, levels of *Pax5* in CLP prior to enhancer deletion were substantially lower (Ct value 29.4) compared to the other five RNAs analyzed (Ct values 22.2–26.5), consistent with its known induction and prominent role later in B cell development. *Ebf1* and *Pax5* expression was not detected in LMPP from Enh(f/f) mice, and *Cebpa* enhancer deletion did not affect LMPP expression of *Izkf1*, *E2A*, *Foxo1*, *Bach2*, or *Pu*.*1* (Fig 2 and S4 Fig). These data suggest that amongst several key B-lineage regulators, the gene encoding *Ebf1* specifically requires C/EBPα for induction, with ~10-fold reduced *Cebpa* leading to ~40-fold reduced *Ebf1* mRNA in CLP.

### C/EBPα binds and activates the *Ebf1* gene via a conserved +280 kb enhancer element

**Fig 3:**
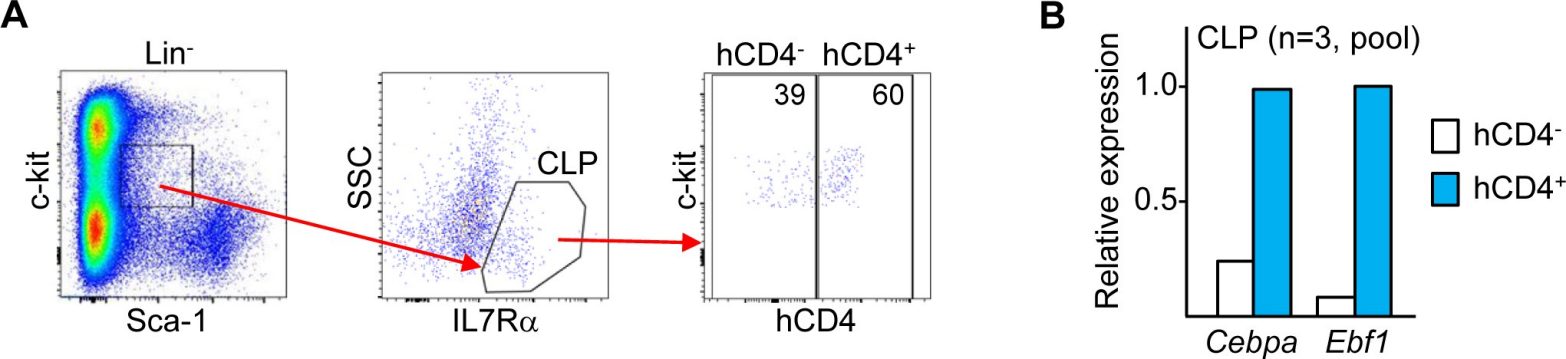
*Cebpa* and *Ebf1* mRNA are both increased in hCD4^+^ versus hCD4^-^ CLP from *Cebpa* +37 kb Enh/Prom-hCD4 transgenic mice. **A**) Lin^-^Sca-1^int^c-kit^int^IL7Rα^+^ CLP from *Cebpa* Enh/Prom-hCD4 mice were sorted into hCD4^+^ and hCD4^-^ subsets. Representative flow cytometry analysis prior to sorting is shown. **B**) Sorted hCD4^+^ and hCD4^-^ CLP from three mice were pooled and then analyzed qRT-PCR for expression of *Cebpa* and *Ebf1*.

We previously evaluated *Cebpa* Enh/Prom-hCD4 mice, in which the +37 *Cebpa* enhancer is linked to an 850 bp *Cebpa* promoter segment to direct expression of a human CD4 transgene, and observed that hCD4 is expressed in approximately one-half of CLP [7]. We sorted Lin^-^Sca-1^int^c-kit^int^IL7Rα^+^ CLP from *Cebpa* Enh/Prom-hCD4 mice into hCD4^+^ and hCD4^-^ populations and pooled cells from three mice to determine *Cebpa* and *Ebf1* RNA expression (Fig 3). Flt3 expression was not used during CLP isolation due to the paucity of Lin^-^Sca-1^int^c-kit^int^IL7Rα^+^Flt3^+^ hCD4^+^ and hCD4^-^ cells. *Cebpa* mRNA levels were 4-fold higher and *Ebf1* mRNA levels were 14-fold higher in hCD4^+^ compared with hCD4^-^ CLP, supporting the idea that C/EBPα directly activates the *Ebf1* gene in a subset of CLP.

**Fig 4:**
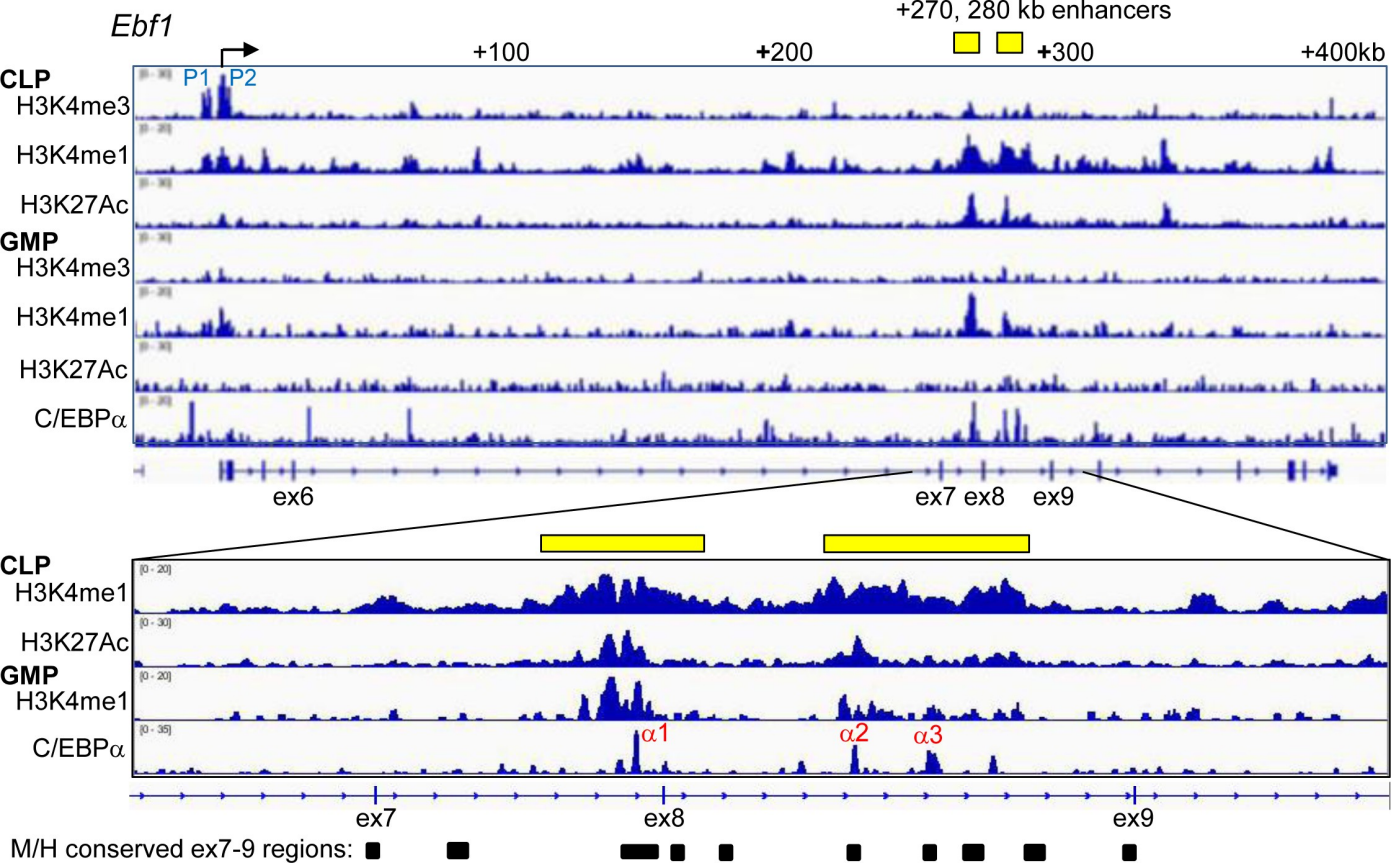
H3K4me3, H3K4me1, and H3K27Ac histone marks at the *Ebf1* locus in CLP and GMP and C/EBPα binding within the *Ebf1* gene in GMP. ChIP-Seq data [28, 29] was visualized with IVG2.0 software (Broad Institute). The region between exon 7 and exon 9 is expanded for a subset of these data. The locations of the two *Ebf1* promoters (P1, P2), potential +270 kb and +280 kb *Ebf1* enhancers (yellow bars), C/EBPα binding peaks in GMP (α1, α2, and α3), and regions between exon 7 and exon 9 conserved between the murine and human *Ebf1* loci (black bars) are indicated.

We next analyzed available ChIP-Seq data [28, 29] for histone marks at the murine *Ebf1* locus in Flt3^+^ CLP and GMP, and also for C/EBPα binding in GMP (Fig 4). The murine *Ebf1* gene is expressed from two promoters [30], as confirmed by the H3K4me3 profile. Presence of both H3K4me1 and H3K27Ac spanning exons 7–9 in CLP indicates existence of active *Ebf1* enhancers centered at approximately +270 kb and +280 kb. In GMP, H3K4me1 marks are still evident at reduced levels in the *Ebf1* enhancer region, and H3K27Ac is absent, suggesting somewhat accessible but inactive chromatin. Three C/EBPα ChIP-Seq peaks are seen in GMP in this region (α1, α2, and α3), and the underlying genomic regions contain blocks of sequence conserved in the human *EBF1* gene, perhaps providing a clue as to relevant C/EBPα-binding sites in CLP.

**Fig 5:**
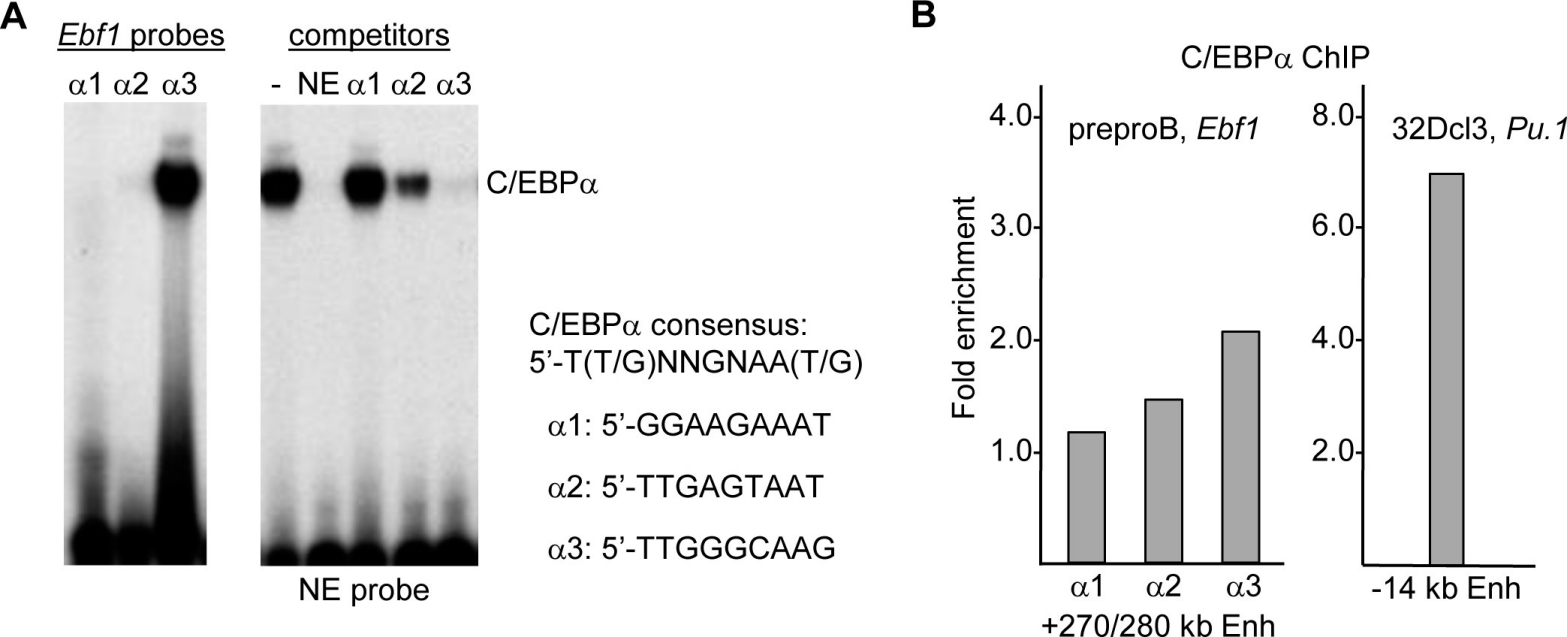
C/EBPα binds a consensus site within the +280 kb *Ebf1* enhancer region. **A**) Double-stranded oligonucleotides containing sites within the α1, α2, and α3 regions that are conserved in the human *EBF1* gene and match or nearly match the C/EBPα consensus binding site were radio-labelled and subjected to gel shift assay using nuclear extracts from 293T cells transiently transfected with CMV-C/EBPα (left). A C/EBPα-binding site from the NE promoter was radio-labelled and subjected to gel shift assay in the absence of competitor or in the presence of 25-fold excess unlabeled NE-C/EBP, Ebf1-α1, Ebf1-α2, or Ebf1-α3 competitor oligonucleotides (right). The location of the gel shift species containing C/EBPα is shown, as is the C/EBPα consensus sequence and the sequence of the predicted α1, α2, and α3 binding sites. **B**) 5 x 10^5^ preproB cells flow-sorted from five wild-type mice were subjected to ChIP for C/EBPα binding in the vicinity of the *Ebf1* +270/280 kb enhancer α1, α2, and α3 peaks, as quantified by dividing fold-enrichment of genomic DNA obtained with rabbit anti-C/EBPα monoclonal antibody versus normal rabbit IgG (left). 10^6^ 32Dcl3 myeloid cells per reaction were analyzed similarly for binding of C/EBPα at the *Pu*.*1* -14 kb enhancer (right).

We identified DNA elements in each of these regions that match or nearly match the C/EBPα consensus site and are 100% conserved in the human *EBF1* locus. These sites, in the context of 30–33 bp double-stranded enhancer sequences, were radio-labeled to similar specific activity and subjected to gel shift assay using nuclear extract from 293T cells transiently transfected with CMV-C/EBPα (Fig 5A, left). C/EBPα interacted with the α3 site strongly and the α2 site very weakly. We also performed a competition assay in which a consensus C/EBPα site from the neutrophil elastase (NE) promoter was radio-labelled and subject to gel shift assay with C/EBPα combined with either no competitor or a 25-fold excess of unlabeled NE, α1, α2, or α3 probes (Fig 5A, right). The NE and α3 oligonucleotides competed strongly with the NE probe, the α2 site competed weakly, and the α1 site did not compete for C/EBPα binding.

To evaluate binding of endogenous C/EBPα in the vicinity of the α1, α2, and α3 sites, we performed ChIP. As preproB are more abundant than CLP and express C/EBPα, we flow sorted this population from five wild-type mice and obtained 10^6^ cells, which we divided evenly between C/EBPα antibody and normal rabbit IgG. As a positive control we conducted ChIP for C/EBPα interaction with the *Pu*.*1* -14 kb enhancer [31] using 10^6^ 32Dcl3 murine myeloid cells per reaction (Fig 5B). Binding of C/EBPα at the endogenous α2 and α3 sites in preproB cells was detected, with binding highest at the α3 site. Presence of cells in the preproB population that express little C/EBPα, including plasmacytoid dendritic cells [27, 32], may reduce evident affinity.

**Fig 6:**
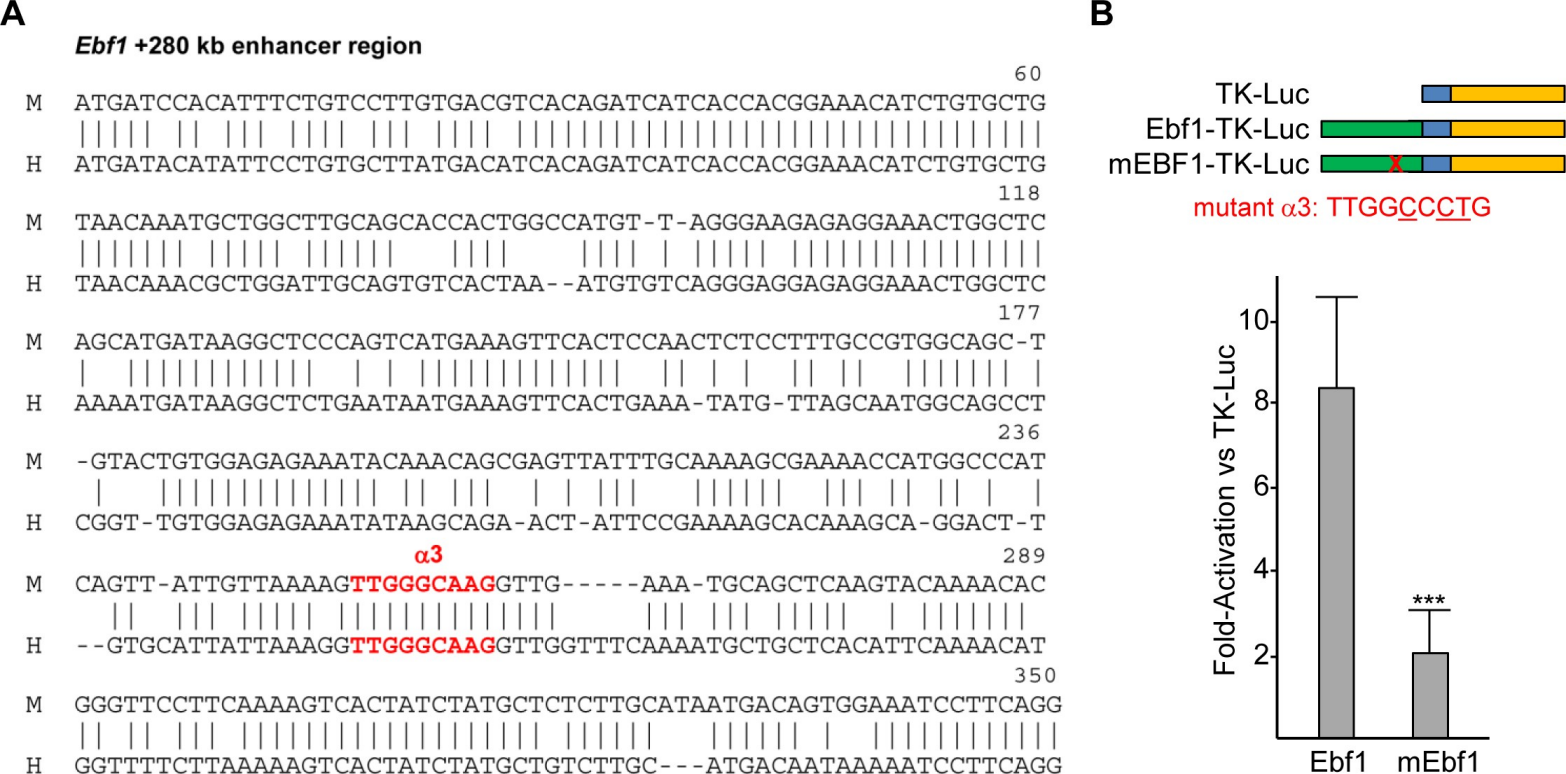
C/EBPα activates an *Ebf1* +280 kb enhancer region via a conserved C/EBPα-binding site. **A**) The sequence of a conserved region within the murine (M) *Ebf1* gene containing the α3 C/EBPα-binding site is aligned with the corresponding region from the human (H) *EBF1* gene. The location of the conserved α3 site is shown. **B**) The TK-LUC, Ebf1-TK-Luc, and mEbf1-TK-Luc reporters are diagrammed. The sequence of the mutant α3 site, with altered bases underlined, present in the mEbf1-TK-Luc reporter is shown (top). 293T cells were transfected with luciferase reporter plasmids, CMV or CMV-C/EBPα, and CMV-βGal in 24 well plates. Two days later, cell extracts were subjected to luciferase and β-galactosidase assays. Reporter activity was calculated as luciferase/βGal activity, and activation by C/EBPα was calculated by dividing activity obtained with CMV-C/EBPα by that obtained with CMV. Activation of Ebf1-TK-Luc and mEbf1-TK-Luc by C/EBPα, relative to activation of TK-Luc, is shown (mean and SD from six determinations).

Alignment of the nucleotide sequence of the 350 bp *Ebf1* +280 kb enhancer region that contains the α3 C/EBPα-binding site with the corresponding region from the human *EBF1* gene reveals that these DNA regions are 78% identical (Fig 6A). The 350 bp murine enhancer segment, or a variant containing point mutations in the α3 site, were positioned upstream of a minimal herpes simplex virus TK promoter, consisting only of a TATAA box and transcription start site, and these were placed upstream the luciferase cDNA, as diagramed (Fig 6B, top). TK-Luc, Ebf1-TK-Luc, and mEbf1-TK-Luc reporters were transiently transfected into 293T cells with CMV or CMV-C/EBPα plasmids; CMV-βGal served as an internal control. Two days later, cell extracts were assayed for luciferase and β-galactosidase activity. C/EBPα activated the Ebf1-TK-Luc reporter 8-fold and the mutant mEbf1-TK-Luc reporter 2-fold, on average, compared to its activation of TK-Luc (Fig 6B). These data support the conclusion that C/EBPα binds and activates the *Ebf1* gene, at least in part via the α3 site located in its +280 kb enhancer region.

## Discussion

We previously found that pan-hematopoietic deletion of the +37 kb *Cebpa* enhancer leads to a profound reduction (greater than 15-fold) in proB, early preB and preB cells, whereas deletion of the enhancer using Mb1-Cre at the proB stage has no effect [11]. We now find that deletion of the *Cebpa* enhancer at the CLP stage of B lymphopoiesis using IL7Rα-Cre leads to ~2-fold reduction in preproB and proB cells and ~3-fold reduction in preB cells, along with 2.4-fold reduced Ly6D^+^ CLP, without inducing the reduced GMP and increased LMPP evident in Enh(f/f);Mx1-Cre mice exposed to pIpC. We demonstrated that IL7Rα-Cre deletes the stop codon in R26-LSL-YFP to enable YFP expression in 65% of multi-potent Ly6D^-^ CLP and in 89% of largely B-lineage-restricted Ly6D^+^ CLP. The kinetics of enhancer deletion by IL7Rα-Cre in CLP, which in one setting required two days to reach maximal effect [22], may allow C/EBPα to induce key target genes prior to *Cebpa* enhancer deletion in a subset B lineage progenitors, reducing the consequence for downstream B lymphocyte precursor formation. Furthermore, constitutive enhancer deletion by IL7Rα-Cre beginning even before birth may engender compensatory mechanisms not operative four weeks after Mx1-Cre-mediated enhancer deletion in adult mice. Nevertheless, the B lineage defect observed in *Cebpa* Enh(f/f);IL7Rα-Cre mice confirms our prior findings obtained using *Cebpa* Enh(f/f);Mx1-Cre and localizes a key point of B lineage regulation by C/EBPα to the CLP stage. IL7Rα-Cre-mediated enhancer deletion also demonstrates that the observed defects in B cell development are not simply an indirect consequence of the reduced GMP or expanded LMPP seen in Enh(f/f);Mx1-Cre mice. Notably, decreased numbers of marrow proB, early preB, and pre B cells were not evident upon *Cebpa* enhancer deletion using Rag1-Cre mice, likely reflecting the reduced propensity for R26-LSL-YFP activation by Rag1-Cre compared with IL7Rα-Cre in CLP.

Analysis of our previous microarray gene expression data reveals 4-fold reduced *Ebf1* in preproB cells consequent to *Cebpa* enhancer deletion, without changes in the expression of RNAs encoding several other transcription factors that mediate B lymphopoiesis. We have now extended this observation by demonstrating a striking, 25- to 50-fold reduction of *Ebf1* in CLP upon *Cebpa* enhancer deletion, without effects on *Pu*.*1*, *Izkf1*, *E2A*, *Foxo1*, or *Bach2*. The level of *Pax5*, a key downstream target of *Ebf1* during B lymphopoiesis [19], was reduced, although its low-level expression in CLP may render the apparent degree of reduction inaccurate. Lack of *Foxo1* reduction despite prior evidence for an EBF1:FOXO1 positive feedback loop [17] indicates that other transcription factors besides EBF1, e.g. E2A or Bach2 [20, 33], are sufficient to induce and maintain *Foxo1* expression in CLP. *Rag1* requires whereas *IL7R*α precedes EBF1 expression [34, 35], consistent with our finding that deletion of the +37 kb *Cebpa* enhancer using IL7Rα-Cre, but not Rag1-Cre, induces a deficiency in downstream B lineage progenitors. Our finding that CLP that express a +37 kb *Cebpa* enhancer/promoter-hCD4 reporter are enriched for both *Cebpa* and *Ebf1* mRNAs, by 4- and 14-fold respectively, further supports the idea that C/EBPα induces EBF1 expression in CLP to contribute to B lymphopoiesis.

The *Ebf1* gene has two promoters separated by 4.4 kb, with the distal promoter activated by STAT5, E2A, and EBF1 and the proximal promoter by ETS1, PAX5, and PU.1 [30]. These promoter regions do not contain DNA elements predicted to bind C/EBPα. We therefore examined available histone mark ChIP-Seq data and identified regions centered at +270 kb and +280 kb that have a pattern of H3K4me1 and H3K27Ac marks in CLP consistent with the presence of active enhancers. These putative intronic *Ebf1* enhancers contain blocks of sequence conserved between the murine and human loci, and we identified a conserved DNA element within one of these regions that binds C/EBPα strongly in gel shift assay. In addition, endogenous C/EBPα in preproB cells binds this region, and mutation of this *cis* element in the context of an *Ebf1* luciferase reporter reduces trans-activation by C/EBPα 4-fold. These data support the conclusion that C/EBPα directly induces *Ebf1* gene expression.

EBF1 binds palindromic DNA sites as a homodimer via a zinc knuckle domain [36, 37]. Mice lacking EBF1 retain preproB but have a profound proB cell deficiency, with B220^+^ B cells lacking immunoglobulin gene rearrangements and expression of multiple proB genes, including *Pax5*, *Mb1*, *Rag1*, and *Rag2*, with normal *IL7Rα* levels and no defects in non-hematopoietic tissues that express EBF1 [35]. Exogenous expression of EBF1 in hematopoietic stem and progenitor cells directs B cell development *in vivo* at the expense of T, myeloid, dendritic, and NK cell formation [38]. Mice lacking E2A display a similar block in proB formation, and transduction of E2A-null fetal liver hematopoietic progenitors with *Ebf1* but not *Pax5* rescues *in vitro* B cell generation [39]. *Ebf1* also rescues B lymphopoiesis in *Pu*.*1*-deficient fetal liver progenitors [40], further supporting its role as a master regulator of B cell development. In addition, EBF1 suppresses *Cebpa* and *Tcf7* expression within progenitors committed to B lymphopoiesis, preventing their induction of myeloid or T lineage genes [41, 42].

**Fig 7:**
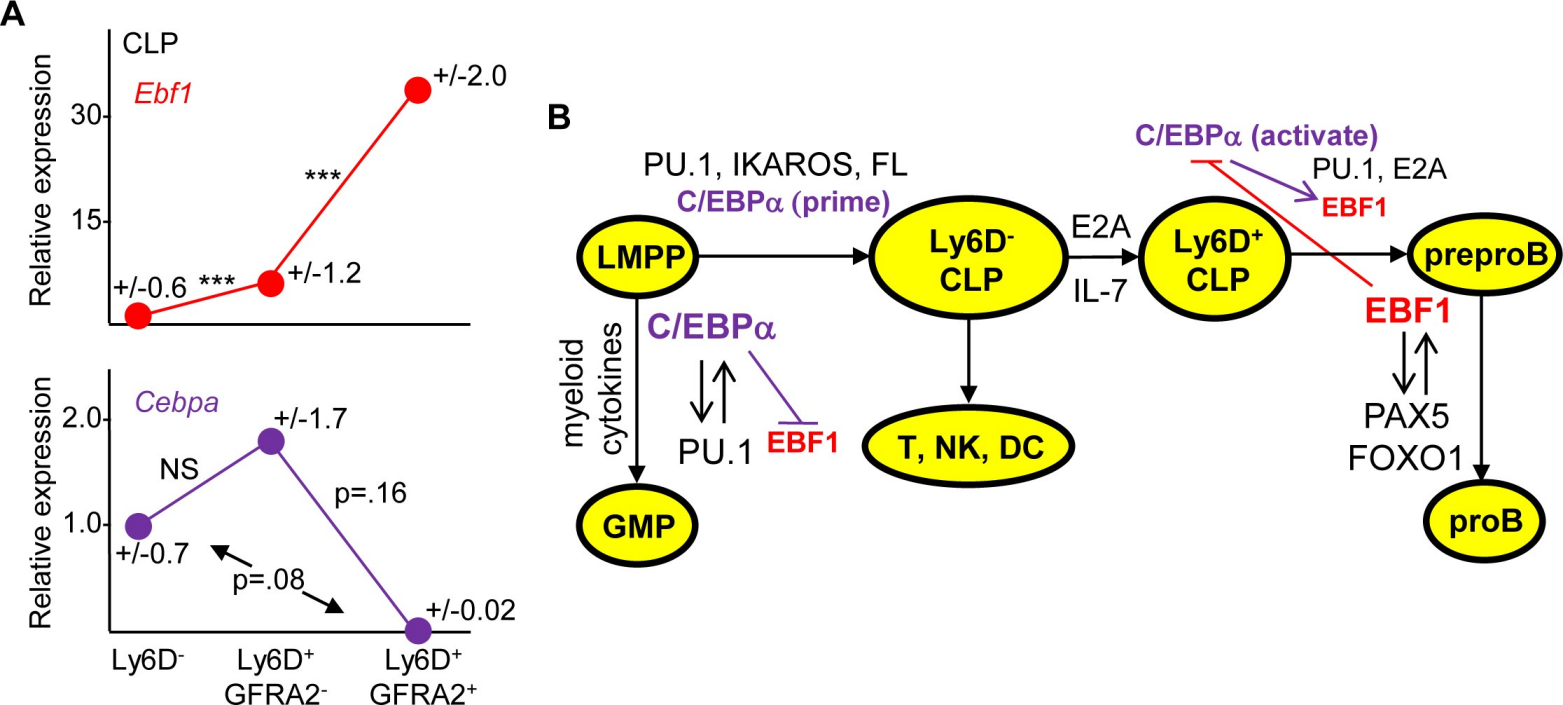
*Cebpa* and *Ebf1* expression in CLP subsets and a model for the role of C/EBPα in B lineage development. **A**) Average RNA-seq reads per million mapped reads [43] from Ly6D^-^ (n = 6), Ly6D^+^GFRA2^-^ (n = 4), and Ly6D^+^GFRA2^+^ (n = 3) CLP, with the mean values for *Ebf1* and *Cebpa* in Ly6D^-^ CLP set to 1.0, are shown. One *Ebf1* outlier value in Ly6D^-^ CLP was excluded. Standard deviations are shown as +/- values. **B**) PU.1, IKAROS, and C/EBPα act in LMPP to prime *Ebf1* and other B lineage genes and, with FL, direct formation and expansion of Ly6D^-^ CLP, which retain T, NK, DC, and B lineage potential. E2A and IL-7 then direct formation and expansion of Ly6D^+^ CLP, wherein C/EBPα directly induces *Ebf1* gene expression. EBF1 activates FOXO1 and PAX5 to direct proB formation and downstream B lineage development while repressing *Cebpa* expression to suppress myelopoiesis. Under the influence of myeloid cytokines, C/EBPα levels increase in LMPP and together with PU.1 directs GMP formation and myeloid lineage development. High levels of C/EBPα may also inhibit *Pax5* expression to suppress B lymphopoiesis in committed myeloid progenitors.

Recent findings indicate that GFRA2 surface expression subdivides Ly6D^+^ CLP into a GFRA2^-^ population without B cell potential and a GFRA2^+^ population having B and T cell potential, with formation of the GFRA2^+^ subset dependent on EBF1 [43]. Quantitative evaluation of RNA-seq data from this study indicates that *Cebpa* levels decrease 70-fold (p = 0.08) comparing Ly6D^-^ with Ly6D^+^GFRA2^+^ CLP, whereas *Ebf1* levels increase 5.9-fold (p<0.001) as Ly6D^-^ CLP progress to Ly6D^+^GFRA2^-^ CLP, and then a further 5.8-fold (p<0.001) as this population progresses to Ly6D^+^GFRA2^+^ CLP (Fig 7A), indicating that *Cebpa* expression precedes *Ebf1* induction in these CLP populations and then decreases.

Single-cell (sc) RNAseq analysis of the Lin^-^c-kit^+^ subset of murine marrow reveals a continuum of progenitor cell states that cluster into populations that include quiescent stem cells and unilineage progenitors, as well as a substantial number of cells that express both lymphoid and myeloid markers (e.g *Flt3* and *Mpo*); scRNASeq analysis of human marrow progenitors reveals a similar bipotent lymphoid-myeloid subset [44, 45]. While our data indicate that C/EBPα acts, at least in part, in CLP to induce *Ebf1*, C/EBPα may play an additional role in earlier bipotent or multipotent progenitors, e.g. to prime *Ebf1* for activation by other regulators. Based on our results and prior findings we propose a model for the role of C/EBPα in B lymphopoiesis (Fig 7B). In this model, C/EBPα, PU.1, and IKAROS prime *Ebf1* and additional B lineage genes in LMPP for later activation and induce progression to the Ly6D^-^ CLP stage. E2A and IL-7 then generate Ly6D^+^ CLP wherein C/EBPα together with PU.1 and E2A induce *Ebf1* gene transcription. EBF1 then enters into a positive feedback loop with FOXO1 and PAX5 to generate proB cells. While Flt3 ligand (FL) and IL-7 signaling may contribute to commitment decisions, e.g. via STAT5 activation, recent findings indicate that they may largely provide proliferative and survival signals [46]. Under the influence of myeloid cytokines, C/EBPα levels increase in LMPP and enter into a positive feedback loop with PU.1 to specify GMP formation [8, 30, 47]. Finally, high levels of C/EBPα may suppress PAX5 to inhibit B lymphopoiesis in committed myeloid progenitors as expression of exogenous C/EBPα in CLP induces their myeloid conversion, with marked PAX5 down-modulation [48].

## Supporting information

S1 Fig Original autoradiograph image used to generate Fig 5A. (PDF)

S2 Fig Flt3^+^ CLP numbers do not change in response to Mx1-Cre-mediated *Cebpa* +37 kb enhancer deletion. Data obtained from Enh(f/f) and Enh(f/f);Mx-Cre mice exposed four weeks earlier to pIpC [11] was re-evaluated for the number of Ly6D^-^ CLP, Ly6D^+^ CLP, and Lin^-^Sca-1^int^c-kit^int^IL7Rα^+^Flt3^-^ cells (designated as Flt3^-^ CLP) in bone marrow from bilateral leg and hip bones (mean and SD from three determinations).
(TIF)

S3 Fig YFP expression in marrow subsets from Enh(f/f);IL7Rα-Cre;R26-LSL-YFP and Enh(f/f);Rag1-Cre;R26-LSL-YFP mice. A) Representative flow cytometry to identify LMPP and CLP subsets from an Enh(f/f);IL7Rα-Cre;R26-LSL-YFP mouse. B) Representative flow cytometry to identify preproB, proB, early preB, and preB marrow cells from an Enh(f/f);IL7Rα-Cre;R26-LSL-YFP mouse. C) Representative flow cytometry evaluating YFP expression in these marrow subsets from Enh(f/f);IL7Rα-Cre;R26-LSL-YFP and Enh(f/f);Rag1-Cre;R26-LSL-YFP mice.
(TIF)

S4 Fig *Cebpa* +37 kb enhancer deletion using Mx1-Cre does not affect *Pu*.*1* levels in LMPP. LMPP from Experiment 2, as described in Fig 2, were analyzed using qRT-PCR for *Pu*.*1* expression.
(TIF)
