# Supplementary material for: C/EBPα induces Ebf1 gene expression in common lymphoid progenitors
Source: PLoS One. 2020 Dec 17;15(12):e0244161. doi: 10.1371/journal.pone.0244161 (PMC7746190; doi:10.1371/journal.pone.0244161)
Supplement: S1 Fig — (PDF) [file pone.0244161.s001.pdf]

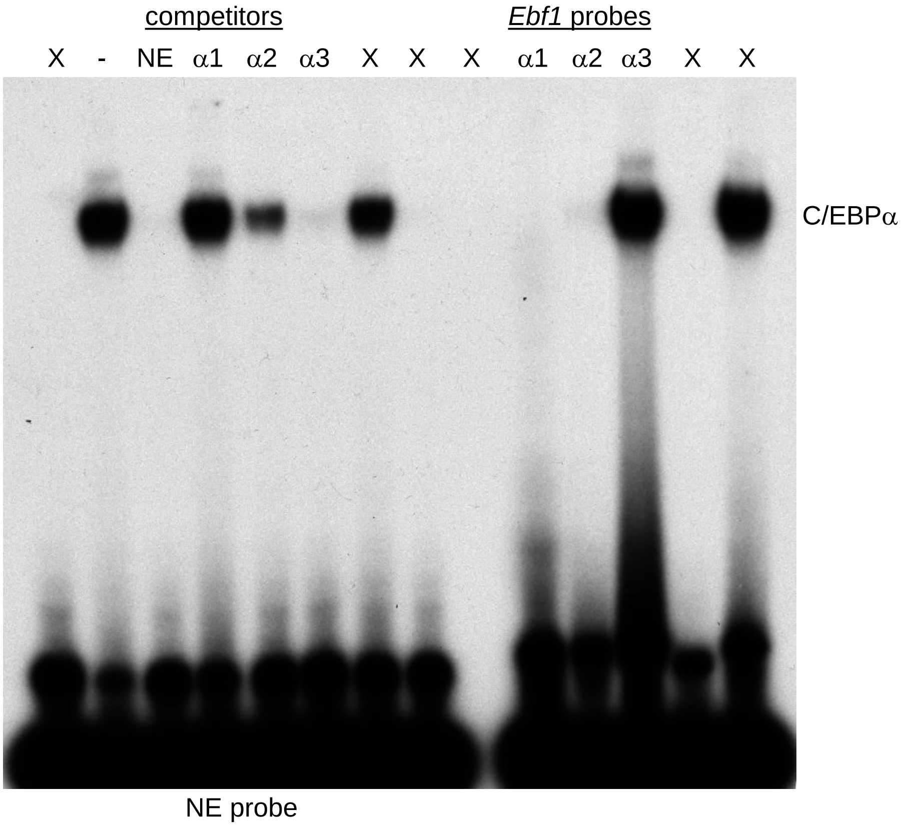

This image was obtained by scanning a gel shift autoradiograph using a GS-900 Calibrated Densitometer (Bio-Rad). Data from this image was used to produce Figure 5A. Lanes not included in the figure are indicated with an X.
