# Supplementary figures and images for: C/EBPα induces Ebf1 gene expression in common lymphoid progenitors
Source: PLoS One. 2020 Dec 17;15(12):e0244161. doi: 10.1371/journal.pone.0244161 (PMC7746190; doi:10.1371/journal.pone.0244161)

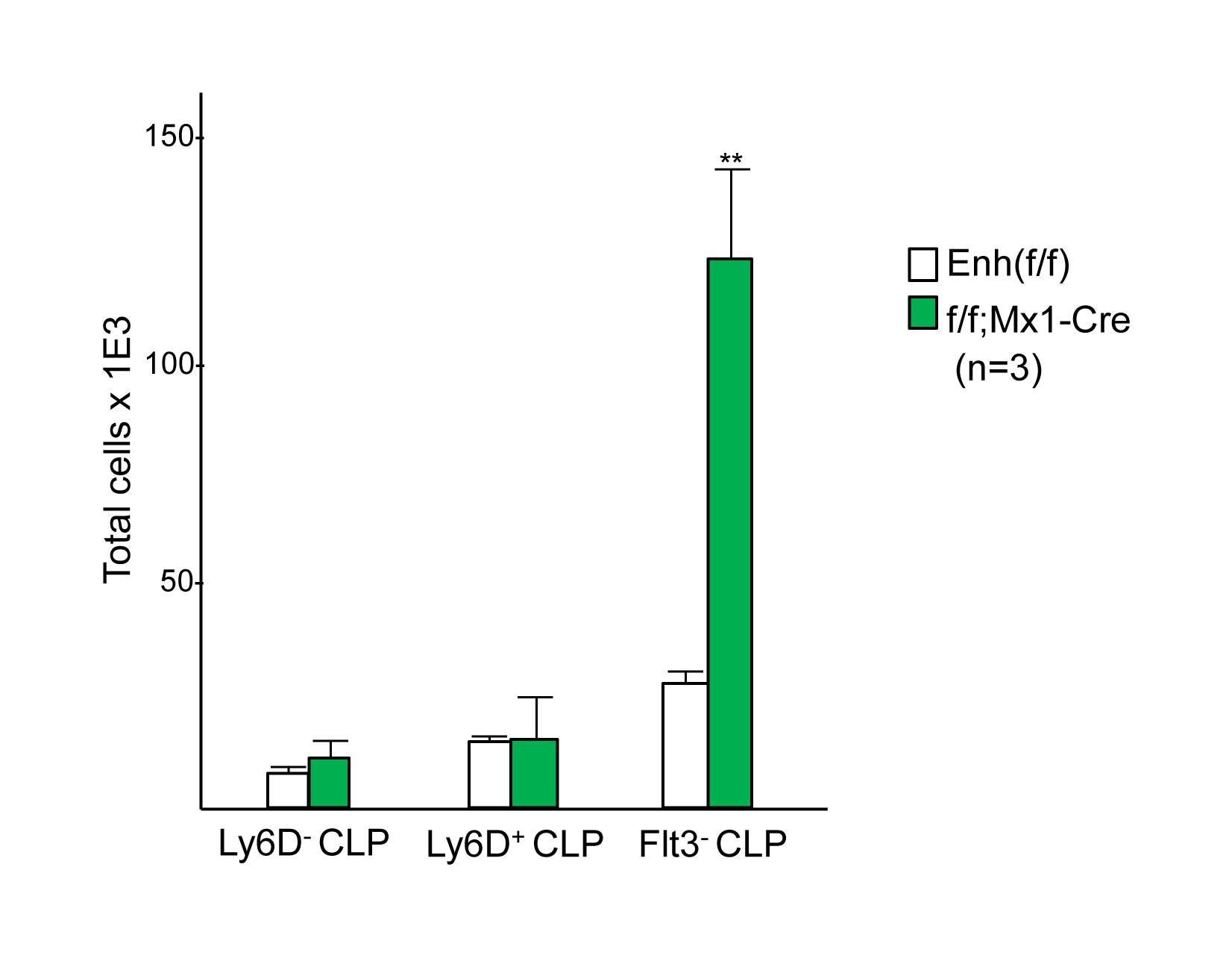

Supplement: S2 Fig — Data obtained from Enh(f/f) and Enh(f/f);Mx-Cre mice exposed four weeks earlier to pIpC [11] was re-evaluated for the number of Ly6D- CLP, Ly6D+ CLP, and Lin-Sca-1intc-kitintIL7Rα+Flt3- cells (designated as Flt3- CLP) in bone marrow from bilateral leg and hip bones (mean and SD from three determinations). (TIF) [file pone.0244161.s002.tif]

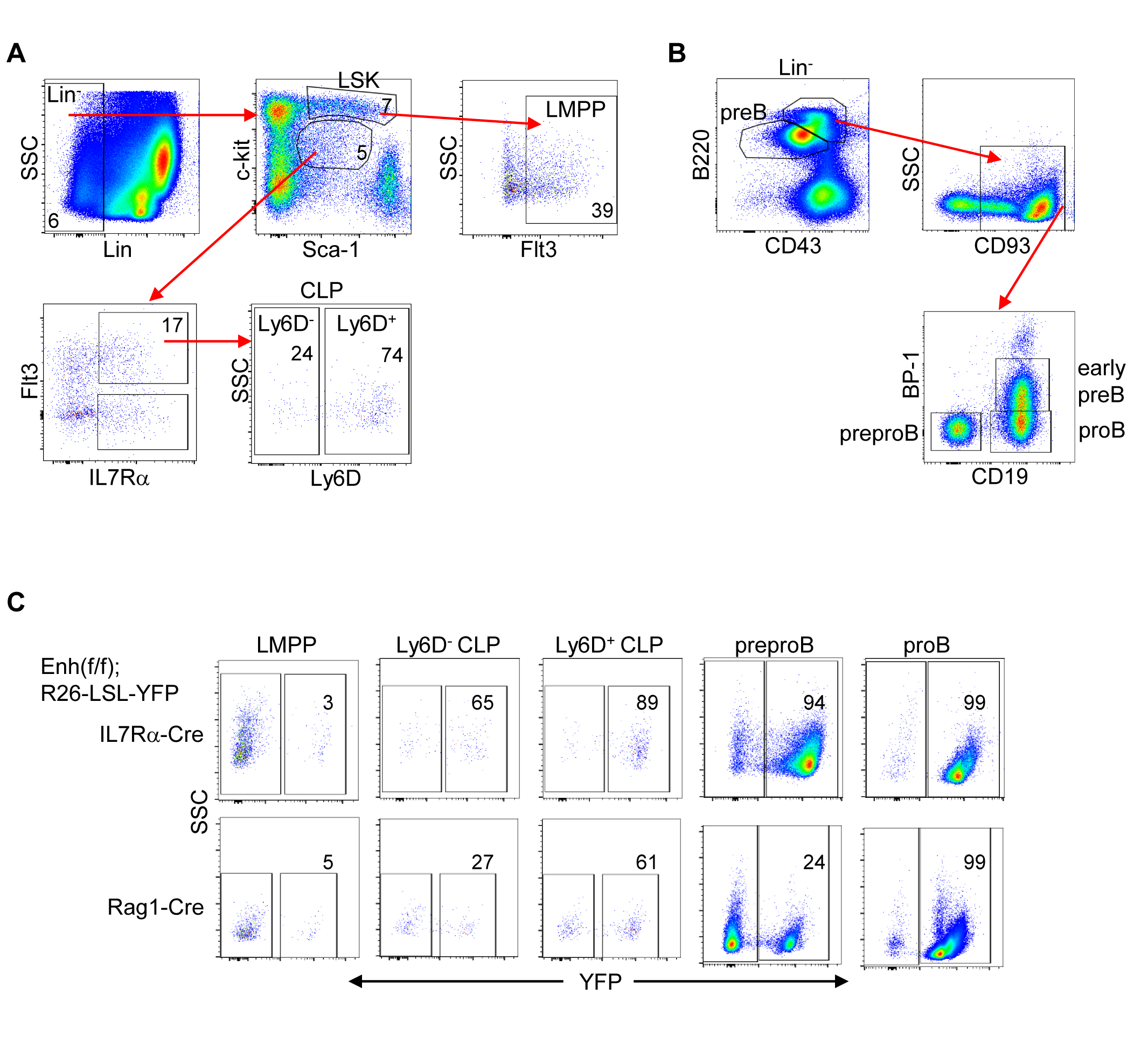

Supplement: S3 Fig — YFP expression in marrow subsets from Enh(f/f);IL7Rα-Cre;R26-LSL-YFP and Enh(f/f);Rag1-Cre;R26-LSL-YFP mice. A) Representative flow cytometry to identify LMPP and CLP subsets from an Enh(f/f);IL7Rα-Cre;R26-LSL-YFP mouse. B) Representative flow cytometry to identify preproB, proB, early preB, and preB marrow cells from an Enh(f/f);IL7Rα-Cre;R26-LSL-YFP mouse. C) Representative flow cytometry evaluating YFP expression in these marrow subsets from Enh(f/f);IL7Rα-Cre;R26-LSL-YFP and Enh(f/f);Rag1-Cre;R26-LSL-YFP mice. (TIF) [file pone.0244161.s003.tif]

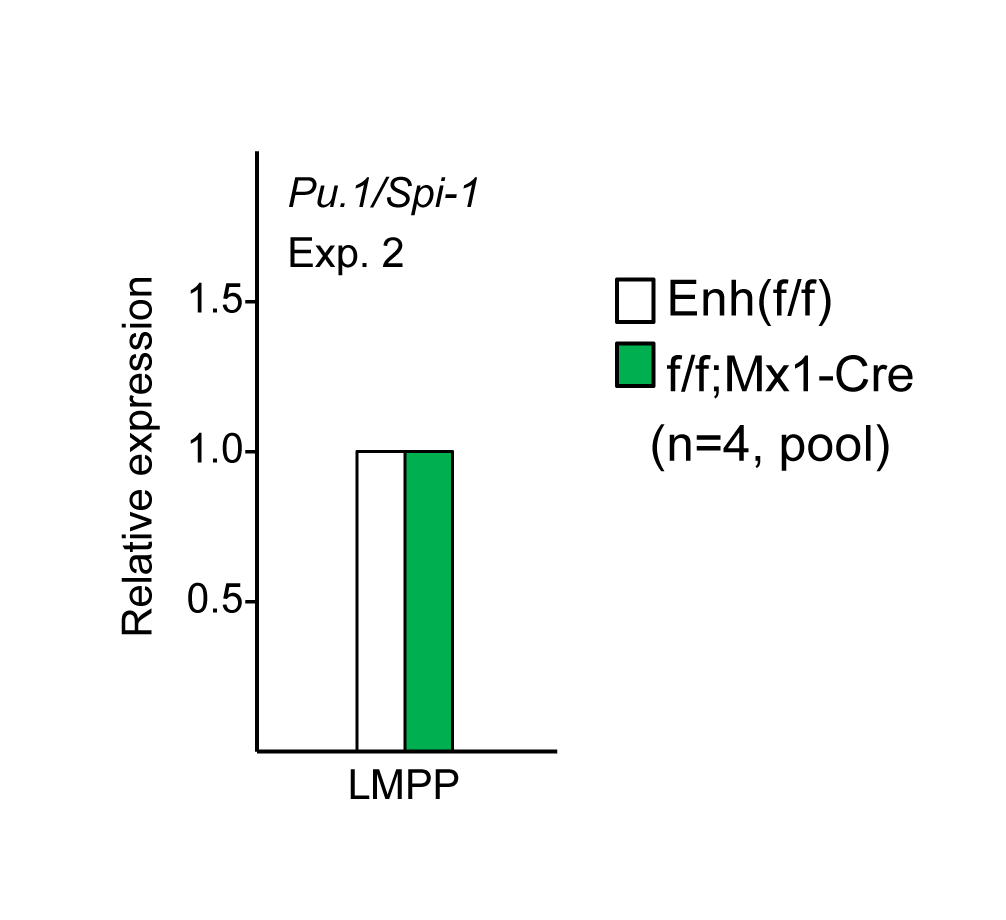

Supplement: S4 Fig — LMPP from Experiment 2, as described in Fig 2, were analyzed using qRT-PCR for Pu.1 expression. (TIF) [file pone.0244161.s004.tif]
